# Supplementary material for: Gluten Induces Subtle Histological Changes in Duodenal Mucosa of Patients with Non-Coeliac Gluten Sensitivity: A Multicentre Study
Source: Nutrients. 2022 Jun 15;14(12):2487. doi: 10.3390/nu14122487 (PMC9230100; doi:10.3390/nu14122487)
Supplement: Supplementary file 1 [file nutrients-14-02487-s001.zip › nutrients-1750724-supplementary.pdf]

Supplementary data/tables

METHOD

Eligibility Criteria: Inclusion criteria for CeD was based on positive serology/HLA and histological abnormalities limited to Marsh I-II and symptomatic/histologic response to a gluten-free diet (GFD). All patients maintained a regular gluten intake (10-15 g/day) for at least 8 weeks before performing the index duodenal biopsy for diagnosis. At least two well-oriented D2 biopsies were examined for each case to achieve measurements for 10 villi and 10 crypts and 10 villi and 10 crypts were measured for each case. Exclusion criteria for CeD were negative serology/HLA tests, lower titre of TTG and no improvement of symptoms and histology on a GFD. The CeD patients with Marsh III lesions were not included in this study.

For NCGS the exclusion of CeD was carried out using the Salerno Expert Criteria[18] with negative coeliac serology in addition to exclusion of wheat allergy and other aetiologies, such as infections or inflammatory conditions. The inclusion criteria for NCGS included patients whose intestinal and extraintestinal symptoms were triggered by gluten exposure and alleviated by gluten withdrawal.
For gluten reintroduction one group underwent the double-blind placebo-controlled gluten challenge as recommended by the Salerno expert criteria[1, 18]. Another group followed an open gluten challenge approach. Patients had full knowledge of consuming a quantity of at least 8g of gluten/day for a duration of 7-21 days in order to reach a threshold for confirming or excluding the diagnosis of NCGS. The clinical features and histologic data of two groups were compared and patients with negative gluten challenge were excluded from study.

Interclass Correlation Coefficient:

In order to validate the quantitative histologic analysis, an inter-observer agreement (IOA) study was planned and a set of 24 anonymized slides comprising well-oriented 8 duodenal biopsies from each group (type 1 CD, NCGS and controls) selected among the original cases included in the study were distributed among the leading expert pathologists (pathology work force comprising AE, VV, AS, PD, GB who are responsible for the design of the histopathological evaluation) and assessed the same histologic morphometric parameters used in the study. An Interclass Correlation Coefficient Analysis was performed on the results to obtain the level of agreement between the observers. Sample size ("number of subjects") for this analysis (i.e.24 cases) was determined using the two parameters: number of observations per subject and the ICC for both 80% and 90% power levels (see reference 37) which ranged from 2 to 15 subjects. The results of Interclass Correlation Coefficient Analysis revealed good

to an excellent agreement on IEL counts per 100 enterocytes, villous height (VH), crypt depth (CrD), PVIIEL and PVEIEL, and a moderate agreement was achieved on eosinophil counts in the lamina propria. Data is presented in Supplementary Table 4.

Suppl Table S1: Contributing centres and countries by number of subjects in each study group

Country	Centre	Control	NCGS	Coeliac	Total
Australia	University of Newcastle, Callaghan,	10	0	10	20
Finland	Tampere University	10	0	10	20
India	All India Institute of Medical Sciences	15	1	9	25
Iran	Shahid Beheshti University of Medical Sciences	10	10	17	37
Italy	University of Perugia	10	0	23	33
Italy	University of Palermo	0	19	2	21
Italy	Desio Hospital	10	10	10	30
Italy	Università di Salerno	10	3	10	23
Italy	Policlinico di Milano	12	16	9	37
Italy	Spedali Civili di Brescia	10	10	11	31
Italy	University of Bologna	4	10	6	20
Romania	Grigore T. Popa University, Iasi	0	3	7	10
Turkey	Ankara University	10	10	10	30
UK	Birmingham City University	5	1	4	10
UK	Luton Dunstable University Hospital	10	4	6	20
UK	Milton Keynes University Hospital	11	10	0	21
UK	University of Glasgow	47	7	61	115
USA	Massachusetts General Hospital	28	56	4	88
USA	University of Chicago	10	0	25	35
USA	Weill Cornell Medicine	10	0	10	20
USA	University of Rochester	10	2	3	15
USA	Brigham & Women's Hospital, Boston	10	0	10	20
USA	Mayo Clinic, Rochester	10	3	4	17
**All**		**262**	**175**	**261**	**698**

Suppl Table S2. Predominant symptoms in three main groups								
**Symptom**	**Controls**		**NCGS**		**Coeliac**		**Total**	
	**N**	**%**	**N**	**%**	**N**	**%**	**N**	**%**
Abdominal Pain	53	20.2	34	19.4	36	13.8	123	17.6
Anaemia	39	14.9	12	6.9	39	14.9	90	12.9
Bloating	15	5.7	22	12.6	25	9.6	62	8.9
Diarrhoea	18	6.9	38	21.7	55	21.1	111	15.9
Dyspepsia	76	29.0	18	10.3	29	11.1	123	17.6
Family Hx of Coeliac	1	0.4	6	3.4	18	6.9	25	3.6
GORD	33	12.6	25	14.3	1	0.4	59	8.5
Weight Loss	8	3.1	6	3.4	23	8.8	37	5.3
Fatigue	0	0.0	7	4.0	7	2.7	14	2.0
Other	11	4.2	7	4.0	24	9.2	42	6.0
**Total**	**262**	**100**	**175**	**100**	**261**	**100**	**698**	**100**

There was a range of gastrointestinal and non-specific symptoms recorded as the predominant symptom at the time of diagnosis for each patient, a summary of those symptoms is shown in Supplementary Table 3.

Suppl Table S3. Main histology and serology parameters in NCGS compared to celiac and controls (Note: all figures are median and interquartile range, unless stated otherwise)				
	**Control**	**NCGS**		**Coeliac** (Marsh I/II)
	**N= 262**	(Marsh 0) **N= 92**	(Marsh I/II) **N= 83**	**N= 261**
**Villus Height (µm)**	900 (667-1112)	617 (549 - 863)	465 (390 - 620)	427 (348 - 569)
***p* value (unadjusted)**	$p<0.001$		$p= 0.069$	
***p* value (adjusted)**	$p<0.001$		$p= 0.176$	
**Crypt Depth (µm)**	222 (158 - 294)	296 (261 - 300)	260 (200 - 296)	269 (182 - 323)
***p* value (unadjusted)**	$p<0.001$		$p= 0.545$	
***p* value (adjusted)**	$p<0.001$		$p= 0.409$	
**Villus Height/Crypt Depth Ratio**	4.0 (2.9 - 5.6)	2.1 (1.8 – 2.4)	2.1 (1.5 – 2.6)	1.9 (1.3 – 2.5)
***p* value (unadjusted)**	$p<0.001$		$p= 0.084$	
***p* value (adjusted)**	$p<0.001$		$p= 0.019$	
**IEL /100EC (Villus)**	14 (8 - 20)	18 (14 - 23)	33 (27 - 44)	40 (31 - 50)
***p* value (unadjusted)**	$p<0.001$		$p= 0.003$	
***p* value (adjusted)**	$p= 0.010$		$p= 0.224$	
**IEL/100EC (Crypt)**	3.0	2.7	6.0	6.0

	(2.0 – 6.0)	(1.0 – 5.0)	(2.9 – 12.0)	(3.0 – 11.7)
***p* value (unadjusted)**	*p*= 0.081		*p*= 0.567	
***p* value (adjusted)**	*p*= 0.135		*p*= 0.160	
**PVIIEL**	0%<br>(0 – 10)	20%<br>(0 – 42)	80%<br>(50 – 100)	80%<br>(50 – 100)
***p* value (unadjusted)**	*p*<0.001		*p*= 0.864	
***p* value (adjusted)**	*p*<0.001		*p*= 0.462	
**PVEIEL**	80%<br>(24 – 100)	50%<br>(26 – 100)	67%<br>(48 – 90)	62%<br>(40 – 83)
***p* value (unadjusted)**	*p*= 0.575		*p*= 0.363	
***p* value (adjusted)**	*p*= 0.637		*p*= 0.937	
**Eosinophil Density**	15<br>(10 -18)	11<br>(6 – 15)	10<br>(4 – 19)	16<br>(7 - 29)
***p* value (unadjusted)**	*p*= 0.0.40		*p*= 0.001	
***p* value (adjusted)**	*p*= 0.244		*p*= 0.109	
**TTG titre**	1.3<br>(0.6 - 2.4)	1.2<br>(0.1 - 3.1)	2.2<br>(1.2 - 3.7)	46<br>(24 - 95)
***p* value (unadjusted)**	*p*= 0.777		*p*<0.001	
***p* value (adjusted)**	*p*= 0.430		*p*= 0.010	

Suppl Table S4: Results of inter-observer agreement (IOA) study

Parameter	ICA Coefficient	Significance	Agreement Level
IEL/100 EC	0.851	p<0.0001	excellent
VH	0.737	p<0.0001	excellent
CrD	0.685	p<0.0001	good
VH/CrD ratio	0.715	P<0.0001	excellent
IEL/villus	0.563	p<0.0001	good
IEL/crypt	0.477	p<0.05	moderate
Eos	0.396	p=0.052	moderate
LPI	0.073	NS	poor
POVWIIEL	0.725	p<0.0001	excellent

ICA: Interclass agreement, VH: Villus Height, EC: Enterocyte, CrD: Crypt Depth, Eos: Eosinophile,

Suppl. Table S4: Interclass agreement between pathologists for histological parameters

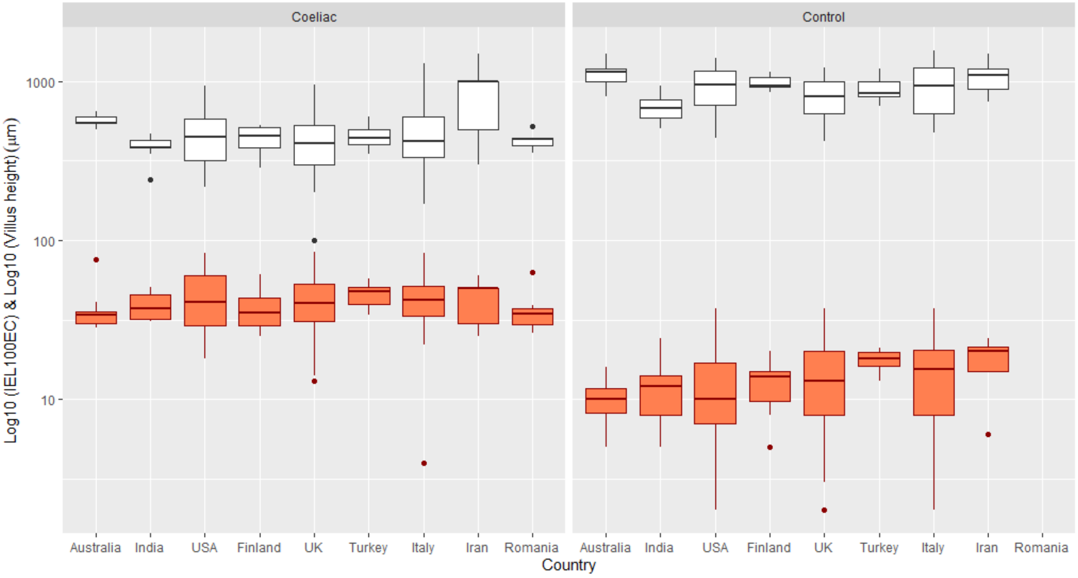

Figure S1. This analysis shows not only the diversity of villus height in different countries but also demonstrate that intestinal villi's are significantly shorter in coeliac disease Marsh I-II compared to controls despite their normal looking appearance.

Villus height Mean

	Australia	Finland	India	Iran	Italy	Romania	Turkey	UK	USA
Coeliac	570	435.5	389.2	888.2	532.4	422.2	447	437.8	461.9
Control	1120	979.0	700.8	1096	928.0	NA	894	818.8	944.8

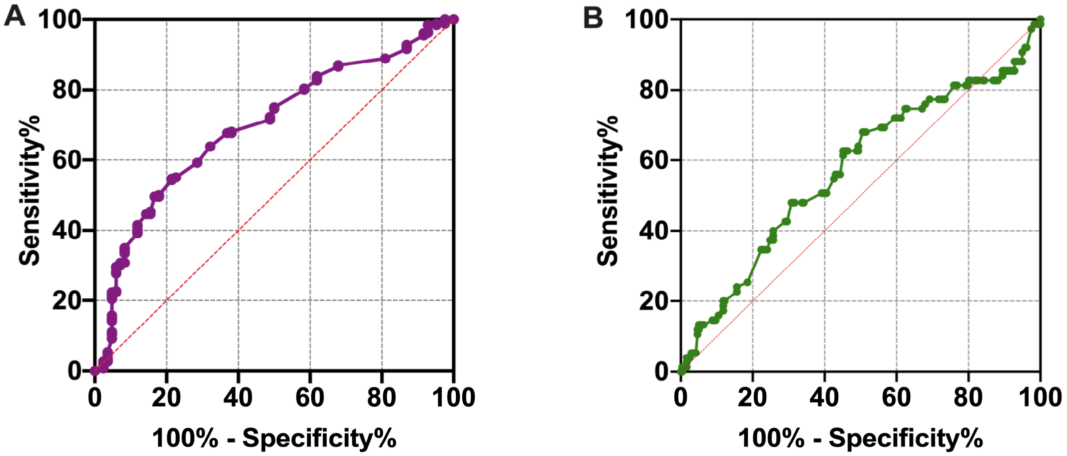

Figure S2. Differential performance of villus IEL density to separate NCGS from CeD and controls groups; **Panel A:** NCGS versus controls, all are Marsh 0; **Panel B:** NCGS versus CeD, all are Marsh I or II.

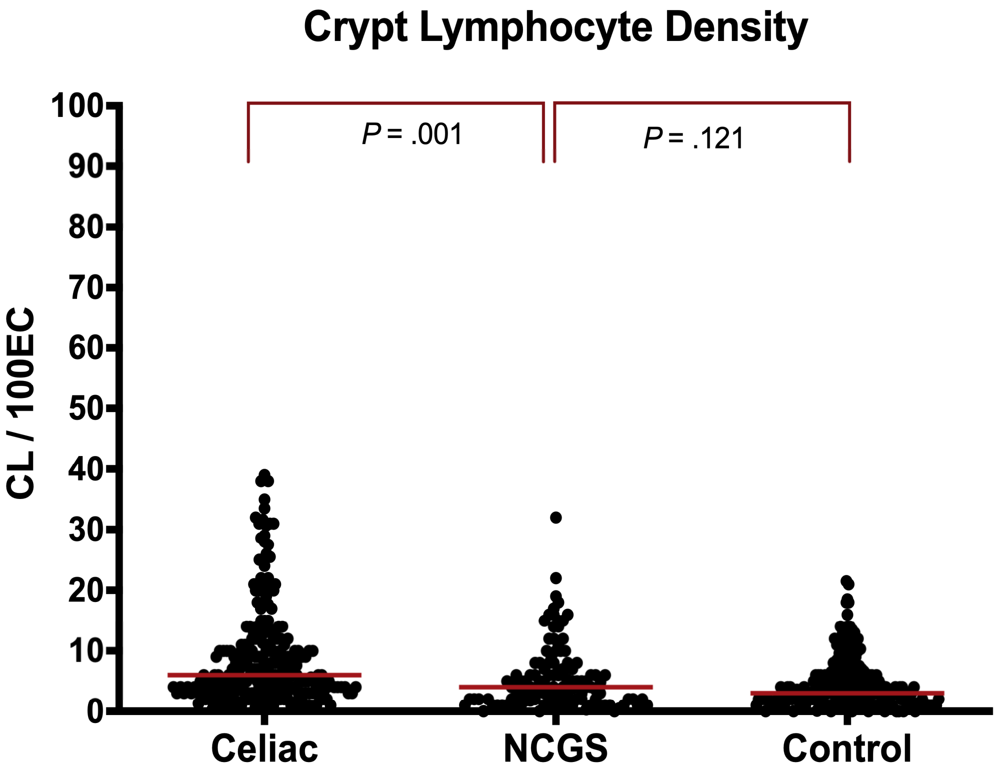

Figure S3. Intraepithelial lymphocytes (IEL) of crypts in NCGS compared with CeD and controls, entire cohort.

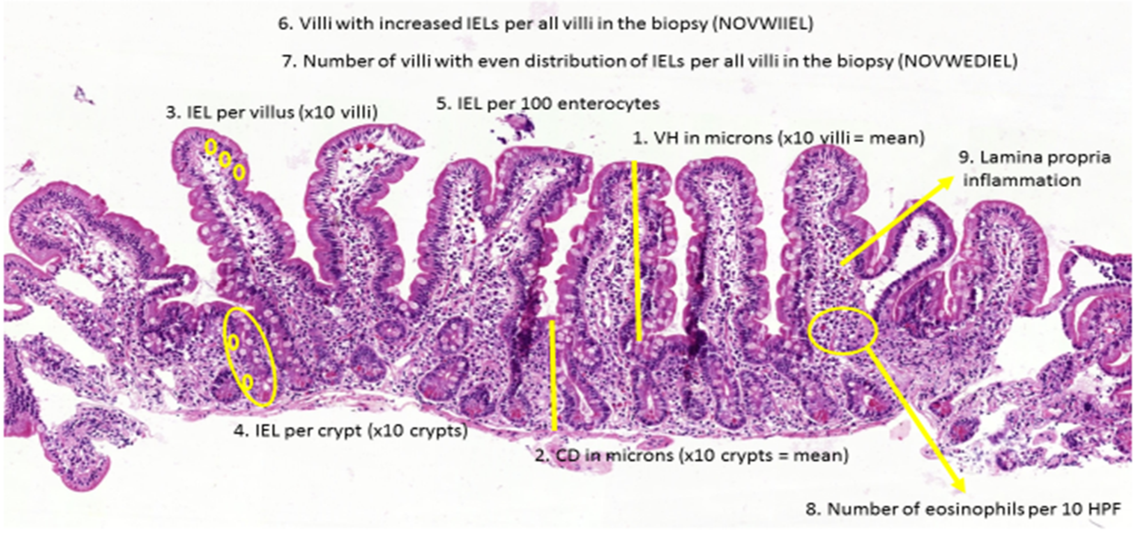

Figure S4. Histomicrograph: Summary of histopathologic evaluation.
